# tarSVM: Improving the accuracy of variant calls derived from microfluidic PCR-based targeted next generation sequencing using a support vector machine

**DOI:** 10.1186/s12859-016-1108-4

**Published:** 2016-06-10

**Authors:** Christopher E. Gillies, Edgar A. Otto, Virginia Vega-Warner, Catherine C. Robertson, Simone Sanna-Cherchi, Ali Gharavi, Brendan Crawford, Rajendra Bhimma, Cheryl Winkler, Nephrotic Syndrome Study Network (NEPTUNE), C-PROBE InvestigatorGroup of the Michigan Kidney Translational Core Center, Hyun Min Kang, Matthew G. Sampson

**Affiliations:** Department of Pediatrics-Nephrology, University of Michigan School of Medicine, Ann Arbor, MI USA; Department of Internal Medicine-Nephrology, University of Michigan School of Medicine, Ann Arbor, MI USA; Department of Medicine, Columbia University College of Physicians and Surgeons, New York, NY USA; Department of Paediatrics and Child Health, University of KwaZulu Natal, Durban, South Africa; NCI, Frederick National Lab for Cancer Research, Molecular Genetics Epidemiology Section, Frederick, MD USA; Department of Biostatistics, University of Michigan School of Public Health, Ann Arbor, MI USA; 3560B MSRB2, 1150 West Medical Center Drive, Ann Arbor, MI 48109 USA

**Keywords:** Microfluidic, PCR, Next-generation sequencing, Variant calling, Accuracy, Support vector machine, Nephrotic

## Abstract

**Background:**

Targeted sequencing of discrete gene sets is a cost effective strategy to screen subjects for monogenic forms of disease. One method to achieve this pairs microfluidic PCR with next generation sequencing. The PCR step of this pipeline creates challenges in accurate variant calling. This includes that most reads targeting a specific exon are duplicates that have been amplified from the PCR step. To reduce false positive variant calls from these experiments, previous studies have used threshold-based filtering of alternative allele depth ratio and manual inspection of the alignments. However even after manual inspection and filtering, many variants fail to be validated via Sanger sequencing. To improve the accuracy of variant calling from these experiments, we are challenged to design a variant filtering strategy that sufficiently models microfluidic PCR-specific issues.

**Results:**

We developed an open source variant filtering pipeline, targeted sequencing support vector machine (“tarSVM”), that uses a Support Vector Machine (SVM) and a new score the normalized allele dosage test to identify high quality variants from microfluidic PCR data. tarSVM maximizes training knowledge by selecting variants that are likely true and likely false variants by incorporating knowledge from the 1000 Genomes and the Exome Aggregation Consortium projects. tarSVM improves on previous approaches by synthesizing variant features from the Genome Analysis Toolkit and allele dosage information. We compared the accuracy of tarSVM versus existing variant quality filtering strategies on two cohorts (*n* = 474 and *n* = 1152), and validated our method on a third cohort (*n* = 75). In the first cohort, our method achieved 84.5 % accuracy of predicting whether or not a variant would be validated with Sanger sequencing versus 78.8 % for the second most accurate method. In the second cohort, our method had an accuracy of 73.3 %, versus 61.5 % for the second best method. Finally, our method had a false discovery rate of 5 % for the validation cohort.

**Conclusions:**

tarSVM increases the accuracy of variant calling when using microfluidic PCR based targeted sequencing approaches. This results in higher confidence downstream analyses, and ultimately reduces the costs Sanger validation. Our approach is less labor intensive than existing approaches, and is available as an open source pipeline for read trimming, aligning, variant calling, and variant quality filtering on GitHub at https://github.com/christopher-gillies/TargetSpecificGATKSequencingPipeline.

**Electronic supplementary material:**

The online version of this article (doi:10.1186/s12859-016-1108-4) contains supplementary material, which is available to authorized users.

## Background

Whole genome sequencing remains a time- and cost-prohibitive method to screen patients for rare and causal genetic variation. Thus, a current strategy involves targeted amplification of only the genomic regions of interest with subsequent focused sequencing. While technological advances have increased the throughput of sequencing platforms, the target enrichment step remains low-throughput and rate-limiting. Increasingly, multiplexed microfluidic PCR assays have been employed to increase throughput of the target enrichment reactions in a rapid and cost-effective manner [1, 2]. As an example, the 48.48 Access Array System (Fluidigm, South San Francisco, California, USA) is a chip-based platform that uses microfluidics to reduce sample requirements and increase scale, enabling multiple PCR amplification reactions to occur simultaneously. With 48 DNA inlets and 48 primer inlets, each chip permits 2300 simultaneous amplification products. To further increase the scale, reactions have been multiplexed with 10 primer pairs per inlet, resulting in a total of 23,000 unique amplification products. Thus, multiplex microfluidic PCR assays permit target enrichment at an increased scale, which can then be integrated with next generation sequencing (NGS) technologies, where the replicated PCR products are sequenced.

A primary goal in data analysis is to maximize the accuracy of variant calling from the targeted sequence data generated. False variant calls can arise from numerous sources, such as errors in base calling, or artifacts that occur when aligning samples’ reads to the reference genome [3]. In addition, multiplexed microfluidic PCR assays have unique factors that can increase potential for false variant calling. First, the polymerase can incorporate incorrect nucleotides during target amplification. The subsequent PCR product, now containing an error, can itself be used as the target for additional rounds of amplification. If the product accumulates in sufficient quantity, the product will be sequenced. This altered sequencing read is now a false positive finding, a technical artifact masquerading as a variant. Second, the process of target amplification for focused sequencing inherently creates multiple duplicate sequencing reads. Compared to whole genome sequencing – where duplicate reads can be removed and the presence of unique sequencing reads increases confidence in variant calling – the presence of duplicates reads that cannot be removed in targeted sequencing strategies can confound the analysis. Together, these limitations necessitate intense scrutiny of the sequence data to minimize false variant calling.

Following multiplexed microfluidic PCR amplification and subsequent sequencing, a common approach to data analysis involves using commercial software for sequence alignment and variant calling [2, 4]. Subsequently, filtering strategies have been developed to improve accuracy of variant calling. While developing a filter, Halbritter and colleagues studied the distributions of allele balance (alternative allele read depth ratio) and alternative allele depths for heterozygotes [2]. They suggested that an allele balance of 20 %--a ratio of alternative alleles to total alleles of >0.2—be required as a filtering mechanism. In addition, they recommended requiring that the alternative allele appear in at least 10 reads (alternative allele depth). After discovering variants meeting this criteria, manual inspection of the alignments is necessary to distinguish between primer artifacts and true variation. Even following careful and labor-intensive filtering strategies, many identified variants are subsequently not confirmed by Sanger sequencing.

Thus, there is a need to develop a more accurate and more fully automated variant calling pipelines when working with microfluidic PCR based NGS studies. In this paper, we describe **tar**geted sequencing Support Vector Machine (“tarSVM”), an open source pipeline based on Genome Analysis Toolkit (GATK) [5] that seeks to achieve this goal. Our pipeline is a synthesis of the ideas from previous studies calling variants from microfluidic PCR coupled with NGS [1, 2], many suggestions from GATK’s best practice filter, and GotCloud’s [3] SVM filtering strategy. Our pipeline trims adapters from reads, aligns to the reference genome, performs variant calling, and uses a novel SVM which models allele balance and features summarized by GATK to perform variant quality filtering. tarSVM is further improved by incorporating knowledge from the 1000 Genomes [6] and the Exome Aggregation Consortium (http://exac.broadinstitute.org) (ExAC) to maximize the number of variants to learn the SVM’s decision boundary. Using two independent cohorts of participants with kidney phenotypes, we constructed this pipeline and compared it to existing and alternative methods. tarSVM was then validated using Sanger sequencing on a third cohort. The software is available on GitHub at https://github.com/christopher-gillies/TargetSpecificGATKSequencingPipeline.

## Results

### Overview

In this study, our goal was to develop a more efficient and accurate filtering pipeline to identify true variants identified by microfluidic PCR followed by next generation sequencing (NGS). To achieve this, we used two existing NGS datasets from cohorts of subjects with kidney phenotypes who had undergone targeted diagnostic screening of disease-associated gene panels using microfluidic PCR + NGS. NGS-based sequence reads from these two existing datasets had previously evaluated using the CLC Genomics Workbench™ (https://www.qiagenbioinformatics.com/) including quality control via manual inspection of the alignments. Variants called with this approach that were also predicted to be pathogenic (including allele frequency < 1 %), then underwent Sanger sequencing for confirmation. Variants confirmed by Sanger sequencing were used as a gold standard to analyze predictions from existing methods and our new approach. We then validated our approach using a third cohort with kidney disease who had existing sequencing data. Additionally, to obtain an external evaluation of the sensitivity of different methods, we used an exome array based technology as a gold standard for which to compare. All analyses were performed on the Variant Call Format (VCF) file generated from our pipeline (see Methods) except where noted otherwise.

### Terminology

In the following discussion, we assume that the ground truth is the set of variants that underwent Sanger sequencing. The below statements also apply if the exome array dataset was used as the ground truth. Please note that we use tarSVM as a placeholder for any of the filtering methods that we evaluate.

The set of variants that underwent Sanger sequencing has two subgroups (1) sites validated by Sanger sequencing and (2) sites not validated by Sanger sequencing.

- A site that is found in the Sanger sequencing data and passed tarSVM is a true positive (TP).
- A site that is validated in the Sanger sequencing data but not identified by tarSVM is a false negative (FN).
- A site not validated in Sanger sequencing data and not identified by tarSVM is a true negative (TN).
- Finally, a site that is not validated in the Sanger sequencing data, but identified by tarSVM is a false positive (FP).
- Sensitivity = TP/(TP + FN), the proportion of Sanger validated sites identified by tarSVM.
- Specificity = TN/(TN + FP), the proportion of sites that were not validated via Sanger that were also rejected by tarSVM.
- False discovery rate = FP/(FP + TP), the proportion of sites identified by tarSVM and underwent Sanger sequencing that were not validated by Sanger.
- Accuracy = as (TP + TN)/(TP + FP + TN + FN), the proportion of all sites that underwent Sanger sequencing that tarSVM predicted correctly.

### Dataset descriptions

The *first cohort* (“NS Cohort”) was comprised of 474 subjects, 413 with nephrotic syndrome (NS) enrolled in either the Nephrotic Syndrome Study Network (NEPTUNE) [7] or C-PROBE and ancestry matched 61 population controls from the 1000 Genomes Project. In this cohort, 21 genes implicated in monogenic NS were amplified using Fluidigm Access Array and sequenced using Illumina NGS instruments. The NEPTUNE cases and 1000G controls have been previously described [8]. The *second cohort* (“CAKUT Cohort”) consisted of 1152 subjects sequenced across 38 genes associated with Congenital Anomalies of Kidney and Urinary Tract (CAKUT). To validate our SVM based-filtering, we applied our approach to a third cohort (“SA Cohort”) comprising 75 South African subjects with NS sequenced across 28 genes implicated in monogenic NS nephrotic syndrome in the same manner as the NS cohort.

### Next generation sequencing statistics

In the NS Cohort, 95 % of samples had a mean depth greater than 157, and 80 % of samples had a mean depth greater than 365. In the CAKUT Cohort, 95 % of samples had a mean depth of at least 296, and 80 % of samples had a mean depth of at least 526. In the SA Cohort, 95 % of the subjects had a mean depth greater than 109, and 80 % of subjects had mean depth of at least 152.

### Accuracy of CLC-based filtering strategy

A variant filtering strategy using CLC Genomics Workbench™ (CLC) paired with manual inspection of potential variants sites yielded a high false discovery rate (FDR). Specifically, in the NS Cohort, (472 sites with sufficient mean read depth) this filtering strategy identified 142 sites that harboring missense, splice variants, or stop gained variants classified as putatively pathogenic and which thus were subsequently were Sanger sequenced. Of these 142 variants, 83 (58 %) were validated with Sanger sequencing and 59 (42 %) failed validation. Overall there was a 42 % FDR. The vast majority of variants were found in the heterozygous state in a single individual (singleton). However for the rare cases where multiple subjects had a variant at a site, we defined a site as true if at least one variant was Sanger confirmed at that site. We also limited our analysis to single nucleotide variants, because of the relatively few indels identified.

Of the 1152 CAKUT subjects, 28 were excluded because of low mean depth. The CLC-Workbench-based analysis identified 371 rare sites of variation that were putatively pathogenic. Of these, 159 were validated with Sanger sequencing (42 %). Thus, the FDR for the CAKUT Cohort was 57 %.

### Sensitivity of tarSVM

We first sought to determine whether our SVM-based strategy was sensitive to detect polymorphic sites (sites that have a non-reference allele on one or more chromosomes) and sites that have a non-reference allele on exactly one chromosome within the target geneset. To do this, we took advantage of the fact that 373 (79 %) NS Cohort participants had been previously genotyped using the Illumina Human Exome-12 v1A (“Exome Chip”). In the Exome Chip array, there were 513 potential variant sites among the 21 genes. In our 373 NS subjects, 311 of these sites were monomorphic (no non-reference variants detected) and 202 were polymorphic. Of the 202 polymorphic sites, 61 had a non-reference allele count of one. We used this independent genotyping dataset as a gold standard so that we could effectively benchmark our method’s sensitivity. Variants with a non-reference allele count of one are rare. Specifically, 69 % of the variants have an allele frequency in the ExAC database less than 10^-3 (Additional file 1: Figure S1).

**Fig. 1:**
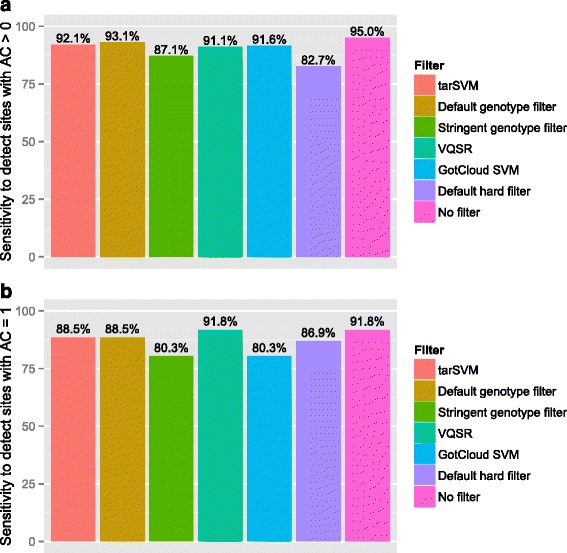
Sensitivity of detecting variant sites in exome chip dataset of 373 subjects. Panel (**a**) shows the sensitivity of detecting sites (*N* = 202) an allele count greater than one in the Exome Chip dataset of 373 subjects using microfluidic PCR for the same subjects. Panel (**b**) shows a similar sensitivity analysis, except it is limited to sites with an allele count of exactly one (*N* = 61). Please note that the “No Filter” bar is an upper bound for all methods except GotCloud SVM. The overall conclusion from these two plots is that tarSVM’s sensitivity is very close to the most sensitive methods for common and rare variants

We evaluated three strategies for their power to detect polymorphic (Fig. 1a) and singleton sites (Fig. 1b). The three strategies were “no filter”, the “default genotype filter”, and “tarSVM” (see Methods). The microfluidic PCR platform, without any filter, identified 95 % of the polymorphic sites on the Exome Chip. The default genotype filter identified 93 % of sites, and tarSVM identified 92 % of all sites. When focusing on singletons, the microfluidic PCR platform with no filter, default, and tarSVM identified 92, 89, and 89 % of singletons, respectively. Thus applying tarSVM to microfluidic PCR-derived NGS data has acceptable sensitivity to detect rare variants.

### Accuracy of tarSVM: comparison to alternative methods

**Fig. 2:**
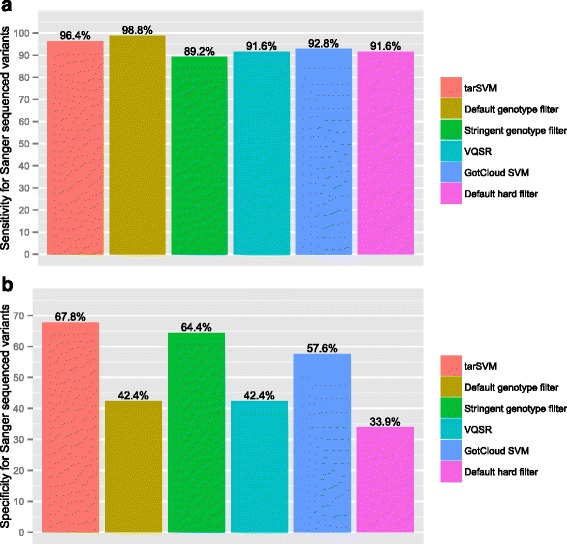
Sensitivity and specificity of filters using Sanger sequenced variants as gold standard for NS Cohort. Panel (**a**) shows the sensitivity of six different filters to detect 142 variants sequenced using Sanger. The default genotype filter has the highest sensitivity, which is not surprising because it was the principal norm for determining whether or not a variant should undergo Sanger sequencing from the CLC Genomics Workbench™. tarSVM is nearly as sensitive as the default genotype filter. Panel (**b**) displays the specificity of six filters. tarSVM is significantly more specific than the default genotype filter

**Fig. 3:**
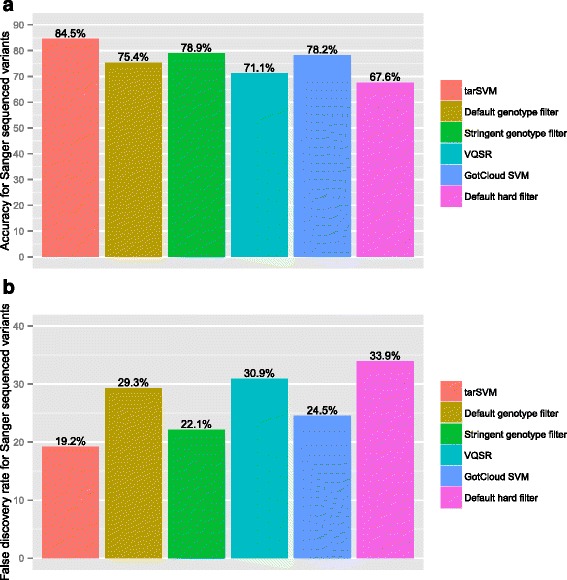
Accuracy and FDR of filters using Sanger sequenced variants as gold standard for NS Cohort. Panel (**a**) shows the accuracy of six different filters for 142 variants sequenced using Sanger. The SVM filter is more accurate than other filter methods. Panel (**b**) illustrates the decreased false discovery rate of tarSVM as compared to other filters

**Fig. 4:**
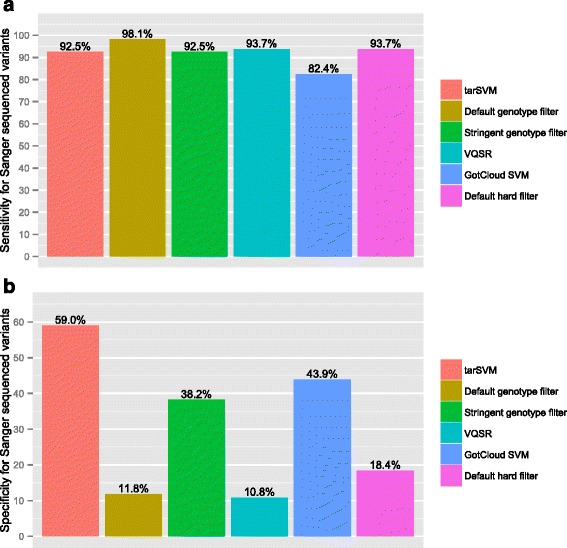
Sensitivity and specificity of filters using Sanger sequenced variants as gold standard for CAKUT Cohort. Panel (**a**) shows the sensitivity of six different filters for 371 variants sequenced using Sanger. The default genotype filter has the highest sensitivity, and tarSVM has comparable sensitivity with other methods. Panel (**b**) displays the specificity of six filters. tarSVM is substantially more specific than other filters

**Fig. 5:**
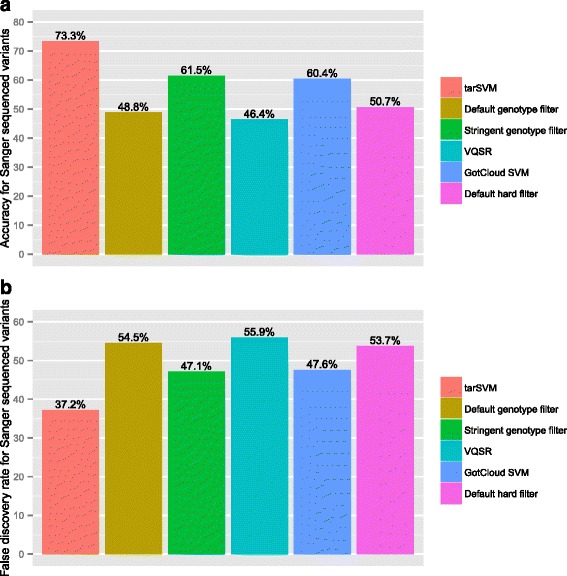
Accuracy and FDR of filters using Sanger sequenced variants as gold standard for CAKUT Cohort. Panel (**a**) shows the accuracy of six different filters for 371 variants sequenced using Sanger. tarSVM is more accurate than other filter methods. Panel (**b**) illustrates the decreased false discovery rate of tarSVM as compared to other filters

We next assessed the sensitivity and specificity of our method in comparison to five other filtering strategies (Figs. 2, 3, 4 and 5). We obtained existing sequence data from microfluidic NGS performed on NS and CAKUT cohort. Variants had been called using CLC-software based method with manual alignment inspection. Called variants from NGS data were confirmed by Sanger sequencing. We considered these Sanger-confirmed. We considered these Sanger variant calls as the gold standard against which we would compare the different methods.

The filtering methods we compared were Variant Quality Score Recalibration (VQSR) [5], GotCloud’s SVM [3], GATK’s best practice hard filter, the default variant quality filter [2], the stringent genotype quality [2], and tarSVM (see Methods for details). First we investigated the sensitivity and specificity using the NS Cohort (Fig. 2). tarSVM was 96.4 % sensitive and 67.8 % specific. Only the default genotype filter had higher sensitivity (98.8 %) than tarSVM. While tarSVM was the most specific filter, the false positive rate was still substantial at 32.2 %.

Figure 3 shows the accuracy and false discovery rate for the same analysis. tarSVM’s accuracy was 84.5 %, which is 5.6 % higher than the stringent genotype filter (the second most accurate filter). Furthermore, it reduces the false discovery rate from 22.1 % (stringent genotype filter) to 19.2 % (tarSVM). Figures 4 and 5 display the same comparison using the CAKUT cohort. The performance of all methods decreased on the CAKUT data. tarSVM’s sensitivity was about 1.1 times lower than the most sensitive filter (98.1 vs. 92.5 %), but its specificity was 1.3 times higher than the second most specific filter (GotCloud). tarSVM’s accuracy was 1.2 times higher than the second most accurate filter (stringent genotype filter). Overall, the accuracy of tarSVM was 73.3 %. tarSVM’s false discovery rate (FDR) was 1.3 times lower than the stringent genotype filter’s FDR.

### Reduction in number of variants to validate using Sanger sequencing

We next sought to determine the magnitude by which utilizing tarSVM would reduce our pool of variants that would subsequently need to undergo Sanger Sequencing. We first applied either the default genotype or tarSVM quality filter to the variants called by GATK coming from the NS and CAKUT cohorts. With these high-quality variants called, we then applied a previously described “default” variant pathogenicity filter to both cohorts, which utilized a combination of allele frequency thresholds and functional prediction scores to protein-altering variants [9].

Table 1 displays a summary of applying the pathogenicity filter to the variant calls sets emerging from the default genotype filter and tarSVM. Overall there were 1054 variants in the NS cohort called by both the default and tarSVM. tarSVM filter called 39 variants not called by the default genotype filter, and the default filter called 209 variants uniquely. Then a previously described pathogenicity filter was applied [10]. As compared to the default genotype filter, tarSVM reduced the number of variants required to validate by 22 % (156 to 121). In the CAKUT cohort, filters were in consensus for 2293 variants. tarSVM called 54 variants not called by the default genotype filter, and the default filter called 1007 variants uniquely. In this cohort, when applying the same pathogenicity filter, tarSVM reduced the number of variants required for validation by 32 % (637 to 430).

**Table 1:** Reduction in number of variants to validate with Sanger sequencing for the NS and CAKUT cohorts

Cohort	Variant quality filter	Total variants	Total variants passing filter	Eligible variants	Pathogenicity filter
NS Cohort	Default	2250	1263	481	156
NS Cohort	tarSVM	2250	1093	408	121
CAKUT Cohort	Default	8812	3300	1564	639
CAKUT Cohort	tarSVM	8812	2347	1135	432

The first column describes the cohort for which the row corresponds. The second column identifies the variant quality filter applied to the dataset. The total variants column refers to the total variants that were called by GATK. The next column shows the number of variants passing a particular variant quality filter for a specific cohort. Eligible variants referrers to all missense and loss of function variants considered for the analysis, excluding frame shift mutations that are considered in the pathogenicity filter. The final column for the pathogenicity filter column displays the number of variants passing having an allele frequency of less than 1 % across all population in the Exome Variant Server, and the variant was either loss of function or predicted to be deleterious by two of MutationTaster, PolyPhen2, and SIFT

### Validation of SVM filter

We next applied our pipeline to the NGS data from the 75-member SA Cohort. After applying tarSVM, we then applied the same pathogenicity filter as used above, which identified 12 rare variants for Sanger sequencing. Of these 12 variants, 11 were validated with Sanger Sequencing, which gives tarSVM a FDR of 8 %. tarSVM filter also correctly predicted common variants implicated in *APOL1*-related kidney disease [11], tarSVM filter thus had an overall FDR of 5 % (1 of 20). An additional three variants were also identified when performing the Sanger sequencing, and tarSVM correctly predicted two of these variants. Thus, the overall accuracy was 20 of 22 or 91 %.

## Discussion

We used a support vector machine approach to develop tarSVM, a fully automated variant quality filtering pipeline that can be applied to targeted next generation sequencing data resulting from microfluidic PCR technology. Existing variant quality filtering strategies designed for whole genome sequencing such as the SVM-based approach in GotCloud’s alignment pipeline [3], Variant Quality Score Recalibration (VQSR) [5], or GATK’s best practice hard filter. GotCloud and VQSR both use machine learning based methods to model true and false variants, and GATK’s hard filter uses specific thresholds for various variant quality metrics to discriminate true from false variants. These approaches do not model microfluidic specific problems well, thus they perform insufficiently on microfluidic PCR data (Additional file 2: Figure S2). Our method achieved similar sensitivity to other variant read quality filtering methods. Importantly, tarSVM substantially increases the specificity as compared to other methods.

In terms of the variant filtration tarSVM is most similar to GotCloud in that they both use hard filters to identify negative training examples and use known variants to identify positive training examples. After selecting the training examples, both methods use an SVM to learn a decision boundary and finally classify all the variants as true or false using this decision boundary. While both algorithms appear similar at this level of abstraction, at a more detailed level of analysis, the differences are manifold and explain the reasons why tarSVM outperforms GotCloud using microfluidic PCR data.

In microfluidic PCR data, a small number of genes are targeted, thus there is a risk of not having enough sites to adequately learn the SVM model. tarSVM addresses this risk by incorporating information from ExAC and dbSNP into the positive training example selection step. GotCloud does not use this information, which is not a problem on the scale of whole genome/exome sequencing. In terms of negative training example selection, tarSVM uses the **n**ormalized **a**llele **d**osage test (NAD) (see Methods) and mean alternative allele depth across heterozygotes to improve the identification of low quality sites. In addition, tarSVM also uses sites that are not labeled “PASS” in ExAC and the 1000G as negative training examples when there is some additional evidence that the site is low quality. tarSVM does not include strand bias scores in the filtration process either which is important for microfluidic PCR since only one strand is being amplified. Additionally, the thresholds for filtering have been tuned primarily using prior information for microfluidic PCR. The NAD and alternative allele depth are also included as features in the model fitting process, which improves the ability of the SVM to learn a versatile decision boundary. Finally, tarSVM applies a genotype-level filter to remove very low quality variant calls (see Supplement for more details).

tarSVM represents a useful step forward in a number of ways. (1) We have created an open source, automated pipeline that transforms raw sequence reads to a filtered VCF file. The pipeline trims adaptors, aligns to the reference genome, and performs variant calling following GATK’s best practice recommendations wherever possible. (2) tarSVM improves positive and negative training example selection over GotCloud for microfluidic PCR data. (3) We created the NAD feature improves the classification of sensitivity and specificity of detecting variants both for hard filtering and for learning the SVM. (4) tarSVM the overall improves the overall accuracy of variant calling. By incorporating knowledge from the 1000 Genomes and Exome Aggregation Consortium and modeling microfluidic-PCR biases such as allele balance, tarSVM more accurately models true and false variants in this application. Thus tarSVM obtains an improved decision boundary, which distinguishes between true and false variants. (5) By fully automating the process of transforming raw sequence reads to a filtered VCF, the tarSVM pipeline removes the dependency of having a user manually reject alignment artifacts as compared the CLC-Workbench approach. Specifically, the pipeline’s read trimming also removes adapters from reads where at least six bases match the adapter starting from the end of the read. This trimming step dramatically reduces the need to manually inspect variants contaminated by adapter sequence. (6) By aligning to the whole genome reduces alignment artifacts because a read can align to its correct genomic coordinates rather than a region of high similarity as compared to the CLC-workbench based approach. (7) Finally, since tarSVM has a higher specificity than other methods, false variants are not carried forward for consideration for potential pathogenicity. This reduces the time and costs associated with Sanger sequencing confirmation. Additional file 3: Supplemental Note #1 and Additional file 4: Supplemental Note #2 provide more details on the impact of these unique aspects on the performance of tarSVM.

In the CAKUT cohort, there was a decreased accuracy of variant calling for all filtering methods tested. We believe this is largely due to the increase number of primers used per inlet on the microfluidic PCR platform. In the CAKUT cohort, 15 primers per inlet were used rather than the 10 primers per inlet for the NS cohort. We posit that unintended interactions between primers within each reaction chamber increased the introduction and amplification of false variants. It is possible that sample size may be affecting the results, but it seems more plausible that the interacting primers are the main cause.

When comparing the call sets emerging from tarSVM and default genotype filters, we found that tarSVM call set is not simply a subset of the default filter. Thus, the default filter may not be capturing all true variants, resulting in false negatives. Because we used variants that were manually inspected and passed the default genotype filter via CLC Genomics Workbench**™**, it is not surprising the sensitivity we calculated was higher for the default genotype filter (the default genotype filter is similar to the filter used with CLC but it starts from the variants called for our pipeline instead). But because of these false negatives, tarSVM’s true sensitivity may not be lower than the default genotype filter. In fact, when performing validation on the SA Cohort, we did not begin with a predefined set of Sanger sequencing variants. In this study, the default genotype filter had sensitivity for the SA Cohort of 90 % (18/20), whereas tarSVM’s sensitivity was 95 % (19/20).

One limitation of this study is that it only investigated the effectiveness of our pipeline on data emerging from the Fluidigm’s Access Array. However, our approach should work for other high-depth targeted sequencing technologies where duplicate reads cannot be removed. The utility of this pipeline is also currently limited to assays where duplicate reads are expected. One possible future direction for this pipeline would be to expand support for duplicate removal, because this will expand support beyond microfluidic PCR-based approaches for targeted rare variant detection (e.g. Molecular Inversion Probes (MIPs) [12, 13].

## Conclusion

Overall tarSVM represents a synthesis of knowledge from high quantity variant call sets and previous filtering strategies. tarSVM achieves similar sensitivity, but substantially improved specificity over other filtering methods for microfluidic PCR data. At the same time, tarSVM is not a panacea for all microfluidic PCR or NGS-based issues. The FDR in the NS cohort improved from 42 to 19 % for our SVM approach, and for the CAKUT cohort the FDR improved from 57 to 37 % FDR with our SVM approach. While these FDRs are much improved, the FDR estimates derived here illustrate the need to continue to use Sanger sequencing to validate variants derived from microfluidic PCR data. However, By automating the process and decreasing the false positive rate, the tarSVM pipeline reduces both sequencing and analytic costs and increases confidence in the variants called in this manner. Finally, it is available as an open source pipeline for read trimming, aligning, variant calling, and variant quality filtering on GitHub at https://github.com/christopher-gillies/TargetSpecificGATKSequencingPipeline.

## Methods

Institutional review board approval was obtained for each of the cohorts described below and participant consent was obtained as well.

### Comparison cohorts

The NS Cohort consisted of 413 subjects with nephrotic syndrome and 61 healthy controls. All subjects had their exons from 21 nephrotic syndrome associated genes amplified on the Fluidigm 48.48 Array Access**™** platform using the same approach as previously published [2]. 10 primer pairs per inlet of the Array Access were used for amplification of exons. 96 subjects were sequenced on the Illumina MiSeq using paired-end sequencing with two times 150 base pairs. The rest of the samples were sequenced using single-end sequencing using the Illumina HiSeq 2500 with reads of length 150 base pairs.

The CAKUT Cohort had 1152 patients and 38 genes associated with CAKUT were amplified using the Fluidigm 48.48 Array Access**™**. The amplicons were sequenced on the Illumina HiSeq 2500 platform using paired-end sequencing (two by 150 base pairs). In order to amplify the large increase in amplicons, the number of primers per inlet of the Array Access was increased to 15.

### Validation cohort

The SA Cohort had 75 subjects from South Africa and with NS, and these subjects were sequenced across 28 genes. The subjects’ exons were amplified using the Fluidigm 48.48 Array Access**™** and the amplicons were sequenced on the Illumina HiSeq 2500 platform using paired-end sequencing (two by 150 base pairs). The number of primers per inlet of the Array Access was 10.

### From raw reads (FASTQ files) to genotypes (VCF file)

Read trimming in the pipeline was performed using cutadapt 1.8 [14]. This step removes the Illumina adapter sequences that can be found at the end of reads and target-specific primers that are found at the beginning and end of reads (prior to the Illumina adapters). Alignment was performed with bwa mem [15, 16]. Read duplicates were not marked for removal because reads are expected to be PCR duplicates. Base recalibration and indel realignment were performed using GATK 3.4. While the pipeline has support for GATK’s HaplotypeCaller, variant calling was performed jointly using GATK’s UnifiedGenotyper, because some known variants were not called using HaplotypeCaller. The result of this pipeline is a Variant Call Format (VCF) file. A Support Vector Machine (SVM) variant quality filter was trained and applied to the VCF file in in order to reduce the false variant calls.

### tarSVM variant quality filter

As an overview, there were four steps (labeled A to D) used in order to use tarSVM filter to classify variants as true or false variants. In step (A), variants were organized into three groups: likely false, unknown and likely true. This classification was achieved through the use of a series of nine hard filters. Variants were trichotomized based on how many filters they failed and whether or not they passed or failed quality control filters in other public datasets. In step (B), we trained a SVM [17] using the likely false and likely true variants, where the variants were modeled using ten site-level summary scores. Step (C) consisted of using the trained SVM to predict the classification of all variants. Step (D) applied genotype-level hard filtering to remove likely low quality variants.

The nine hard filters applied to each SNP in step (A) were: (1) The quality by depth (QD) filter, which is the variant quality normalized by the depth of non-homozygous reference samples, requires a minimum score of 3; (2) the root mean square of mapping quality (MQ) filter requires a score of at least 50; (3) the mapping quality rank sum test (MQRankSum) filter requires a score between −3 and 10; (4) the read position rank sum test requires a score of at least −20; (5) the mean allele balance (ABHet) filter, which is the alternative read depth divided by the total read depth for a site across heterozygotes, requires a score between 20 and 80 %; (6) the mean alternative allele read depth for heterozygotes (AD) filter requires a value of at least 10; (7) the base quality rank sum test (BQRankSum) filter requires a score of at least −40; (8) the call rate filter, which is the proportion of samples called at a particular site, requires a value of 80 %; and (9) the normalized allele dosage (NAD) test for heterozygotes filter required a score of at most 5. The NAD null hypothesis is that that the allele balance is 50 % and the alternative hypothesis is that the allele balance is not 50 %. The test is an exact binomial test and is in the PHRED scale normalized by the alternative allele depth because for high depth the p-values are smaller for the same allele balance.

The scores for most of these filter’s were based on the GATK best practice recommendation’s, however, the scores have been partially adjusted to be more or less stringent based on the distribution of scores empirically observed across the NS Cohort and the CAKUT Cohort.

If a variant failed three or more of these filters, or its call rate was less than 50 %, then it was marked as likely false. If a variant failed two of these filters, we checked whether the variant passes quality control as reported in the 1000 Genomes phase 3 data (ftp://ftp.1000genomes.ebi.ac.uk/vol1/ftp/release/20130502/supporting/site_assessment). If it passed in the 1000 Genomes, we marked the variant’s group as unknown and if it failed we marked it as likely false. We then did the same task for ExAC r0.3 (ftp://ftp.broadinstitute.org/pub/ExAC_release/release0.3/). Finally we checked if the variant appears in dbSNP b138. If it was not present, we marked the variant as likely false. Otherwise, we marked the variant’s group as unknown.

If a variant failed one filter, then we gave the variant its status from the 1000 Genomes or ExAC r0.3. The 1000 Genomes data was given precedence over ExAC r0.3. When it was not present in either, we marked the variant as unknown. When a variant passed all filters, then we gave the variant its filter status from the 1000 Genomes or ExAC r0.3. If it was not present in either, then we checked dbSNP b138, and if it was present it was marked as likely true, otherwise it was marked as unknown.

In step (B) we trained the SVM using likely true and likely false variants. However, some scores were missing when a variant’s callrate was less than 10 % (GATK did not annotate some scores in the VCF) or the site had no heterozygotes (ABHet, NAD, and mean alternative allele depth for heterozygotes could not be calculated). For sites without heterozygotes, we used QD to calculate ABHet using regression-based imputation. We then used the mean alternative allele depth for homozygotes and the imputed ABHet value to calculate a pseudo mean reference allele depth. This information was then used to calculate the NAD for the site without heterozygotes. All other scores were imputed with k-nearest neighbors (k-NN). First we inverse normalized each score and second we applied k-NN with k = 10 to impute missing data using the R package “impute.” After imputation, we trained the SVM using only the sites that were likely false or likely true. The features that we used to model variants were call rate, QD, RPRankSum, ABHet, AD, NAD, inbreeding coefficient, haplotype score, MQRankSum, and BaseQRankSum. We trained the SVM using the R package “e1071” which uses LIBSVM [18].

Step (C) consisted of predicting the class of each variant either “PASS” or “FILTERED”. The trained SVM predicted the class of all variants. Thus some variants labeled as likely false could be predicted as “PASS” by the SVM, and some variants labeled as likely true could be predicted as “FILTERED” by the SVM.

Finally, in step (D), we performed genotype quality filtering. This consisted of setting variants for subjects to be missing if they failed certain criteria. In particular, for heterozygous variants, we required the genotype quality to be at least 40, the AD to be five and the ABHet to be 10 %. For homozygous alternative subjects we required the genotype quality to be at least 40 and the AD to be at least 5.

### VQSR filter

VQSR is a Gaussian mixture model designed to model true and false variants [5]. We used HapMap 3.3 hg19 sites and 1000 Genomes Omni 2.5 sites, ExAC r0.3 and 1000 Genomes phase 3 to model true variants using the same features as the SVM. Also we limited the number of Gaussians to 3.

### Default hard filter

This is the default GATK best practices hard filter (https://www.broadinstitute.org/gatk/guide/best-practices; accessed 7/13/2015) that rejects sites if Quality by depth (QD) is less than two or Fisher Score (FS) is greater than 60 or root mean square of mapping quality (MQ) is less than 40 or mapping quality rank sum test (MPRankSum) is less than −12.5 or read position rank sum (RPRankSum) is less than −8.

### GotCloud SVM

For this method we used the BAM (Binary Sequence Align Map) files generated from our pipeline and used GotCloud’s variant calling pipeline with the WGS_SVM option set to true, which was recommend for targeted sequencing [3].

### Default genotype filter

This filter required the alternative allele read depth for a variant for a subject to be at least 10 and if the subject was homozygous for this variant, then it required the allele balance to be at least 20 % [2]. This filter is very similar to the filter used with CLC Genomics Workbench™ approach, except the thresholds are strictly enforced. This is in contrast to the CLC approach where variants were evaluated on a case-by-case basis so some variants were included that did not meet the filtering criteria. Also this filter was applied to the VCF file generated from our pipeline, and not CLC Genomics Workbench™ analysis pipeline.

### Stringent genotype filter

This filter required the alternative read depth for a variant for a subject to be at least 30 and if the subject was homozygous for this variant, then it required the allele balance to be at least 20 % [2].

### Pathogenicity filter

We examined single nucleotide variants. Functional annotation was performed using SNPEff 3.5 [19], all nonsynonymous variants were annotated using dbSNFP 2.5 [20]. The pathogenicity filter required variants to have an allele frequency less than 1 % in European Americans and African Americans in the Exome Variant Server (http://evs.gs.washington.edu/EVS/accessed March 2015). The variant had to be either missense, stop gained, start lost, or a splice mutation. If a variant was missense, it had to be predicted to be deleterious in two of three of MutationTaster [21], SIFT [22] and PolyPhen2 [23]. When multiple predictions were present for a variant in dbNSFP, the worst prediction was selected.

## Abbreviations

CAKUT, Congenital anomalies of the kidney and urinary tract; MIP, Molecular inversion probes; NEPTUNE, Nephrotic Syndrome Study Network; NS, nephrotic syndrome; SA, South African; SVM, Support vector machine; tarSVM, targeted sequencing support vector machine.

## Additional files

Additional file 1: Figure S1. Allele frequency of variants with AC = 1 in Exome Chip. The log10 allele frequency from the ExAC r0.3 database of variants from the Exome Chip with an allele count of one in our cohort is shown. 69 % of the variants have an allele frequency less than 10^-3. (PPTX 66 kb) Additional file 2: Figure S2. Comparison of quality by depth (QD) from CAKUT and ExAC. Sites that were filtered out of ExAC (VQSR filtering) are shown in red. Sites that were marked as “PASS” in ExAC are shown in gold. Sites that were Not Sanger validated are shown in green. Sites that were Sanger Validated are shown in teal. Sites filtered by tarSVM are shown in blue. Sites that passed tarSVM are shown in purple. Quality by depth is correlated with mean allele balance, as is being used as a proxy for it. It is clear that there is a very clear separation between variants that are filtered by tarSVM and variants that pass tarSVM. Most of the variants filtered by tarSVM have a much lower quality than the pass variants. Variants that are Sanger validated are stronger correlated with variants that pass tarSVM. Variants that are labeled “PASS” in ExAC have a higher variant quality that the microfluidic data. The filtered variants in ExAC have a more flat distribution that those filtered by tarSVM. It is important to note, the variants that underwent Sanger sequencing were selected because they had the characteristics of true variants. This is why there is so much overlap between the distributions for Sanger validated variants and Not Sanger validated variants. (PPTX 121 kb) Additional file 3: Supplemental Notes #1. (DOCX 42 kb) Additional file 4: Supplemental Notes #2. (DOCX 50 kb)
