# Supplementary figures and images for: tarSVM: Improving the accuracy of variant calls derived from microfluidic PCR-based targeted next generation sequencing using a support vector machine
Source: BMC Bioinformatics. 2016 Jun 10;17:233. doi: 10.1186/s12859-016-1108-4 (PMC4902911; doi:10.1186/s12859-016-1108-4)

## Slide 1
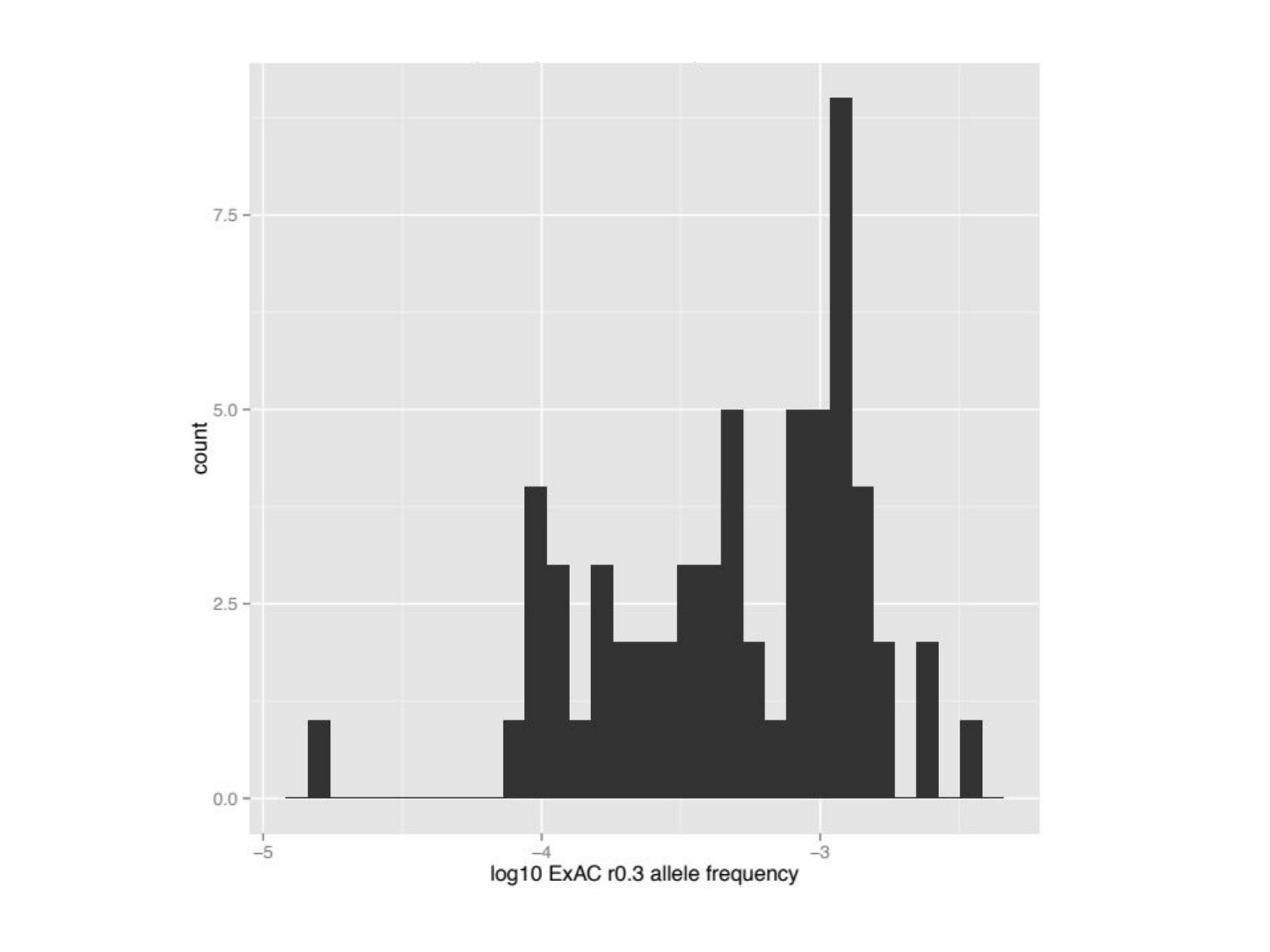

Supplement: Additional file 1: Figure S1. — Allele frequency of variants with AC = 1 in Exome Chip. The log10 allele frequency from the ExAC r0.3 database of variants from the Exome Chip with an allele count of one in our cohort is shown. 69 % of the variants have an allele frequency less than 10^-3. (PPTX 66 kb) [file 12859_2016_1108_MOESM1_ESM.pptx]

## Slide 1
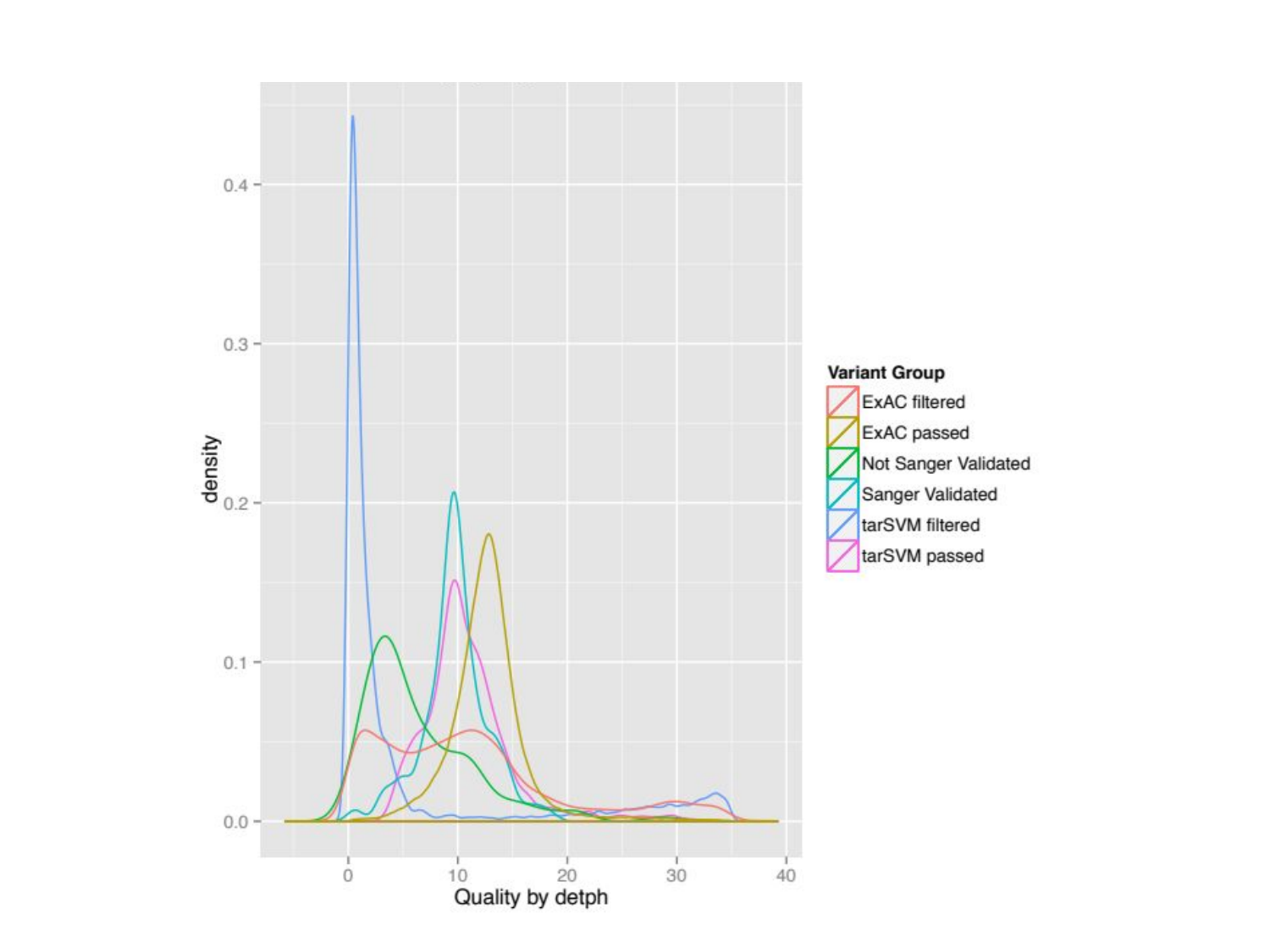

Supplement: Additional file 2: Figure S2. — Comparison of quality by depth (QD) from CAKUT and ExAC. Sites that were filtered out of ExAC (VQSR filtering) are shown in red. Sites that were marked as “PASS” in ExAC are shown in gold. Sites that were Not Sanger validated are shown in green. Sites that were Sanger Validated are shown in teal. Sites filtered by tarSVM are shown in blue. Sites that passed tarSVM are shown in purple. Quality by depth is correlated with mean allele balance, as is being used as a proxy for it. It is clear that there is a very clear separation between variants that are filtered by tarSVM and variants that pass tarSVM. Most of the variants filtered by tarSVM have a much lower quality than the pass variants. Variants that are Sanger validated are stronger correlated with variants that pass tarSVM. Variants that are labeled “PASS” in ExAC have a higher variant quality that the microfluidic data. The filtered variants in ExAC have a more flat distribution that those filtered by tarSVM. It is important to note, the variants that underwent Sanger sequencing were selected because they had the characteristics of true variants. This is why there is so much overlap between the distributions for Sanger validated variants and Not Sanger validated variants. (PPTX 121 kb) [file 12859_2016_1108_MOESM2_ESM.pptx]
