# Supplementary material for: tarSVM: Improving the accuracy of variant calls derived from microfluidic PCR-based targeted next generation sequencing using a support vector machine
Source: BMC Bioinformatics. 2016 Jun 10;17:233. doi: 10.1186/s12859-016-1108-4 (PMC4902911; doi:10.1186/s12859-016-1108-4)
Supplement: Additional file 3: — Supplemental Notes #1. (DOCX 42 kb) [file 12859_2016_1108_MOESM3_ESM.docx]

**Supplementary Note #1**: Influence of tarSVM initial variant classification from hard filtering on classification performance:

**Table S1**: Influence of hard filtration on tarSVM for NS Cohort

**Table S2**: Influence of hard filtration on tarSVM for CAKUT Cohort

**Supplementary Note #1: Influence of tarSVM initial variant classification from hard filtering on classification performance:**

One important question about the performance of tarSVM is: How does the hard filtration step of tarSVM affect the training and classification performance of tarSVM? To answer the question of the influence of the hard filtration on the downstream SVM training, we defined a few different hard filters and assessed how they affect the SVM filtration. The first filter, which we call the “Simplified” filter, starts off by marking all variants as being “Unknown”. Next it marks likely true and likely false variants as follows:

Mark a variant as likely true if the variant meets any condition below

1. The variant is found in ExAC Browser (http://exac.broadinstitute.org/) and is marked as a “PASS” site.
2. The variant is found in 1000 Genomes (http://www.1000genomes.org/) and is marked as a “PASS” site.

Mark a variant as likely false if the variant meets any condition below

1. Mean alternative allele balance < 0.2
2. Mean alternative allele depth across heterozygotes < 10
3. The callrate is < 0.5
4. The variant is found in ExAC and is not marked as a “PASS” site.
5. The variant is found in 1000 Genomes and is not marked as a “PASS” site.

We then modified this filter by removing one condition at a time, for example removing ExAC and assessing what this did to the hard filtration and SVM filtration in terms of sensitivity and specificity. We also defined a “Simplified relaxed” filter that set the mean alternative allele balance < 0.1 and the mean alternative allele depth across heterozygotes to be < 5. The final filter we used was the normalized allele dosage test < 4 as the only filter. The SVMs were all trained with the same parameters as mentioned in the paper.

The results of this analysis can be found in Table S1. Please note that the hard filter sensitivity and the hard filter specificity were calculated only on the variants for which the filter made a prediction. Recall that the hard filter classifies variants into three groups “unknown” “likely true”, and “likely false”. Variants in the unknown category made no contribution to the hard filter sensitivity or specificity.

**Table S1: Influence of hard filtration on tarSVM for NS Cohort**

| Filter | Hard filter  sensitivity | Hard filter  specificity | Number of positive examples | Number of negative  examples | Number of unknown examples | SVM  sensitivity | SVM  specificity |
| --- | --- | --- | --- | --- | --- | --- | --- |
| tarSVM standard | 89.2% | 93.3% | 881 | 1025 | 253 | 96.3% | 66.1% |
| Simplified | 80% | 93.1% | 846 | 1089 | 224 | 96.3% | 71.2% |
| Simplified minus ExAC | 93.5% | 100% | 741 | 1023 | 395 | 96.3% | 62.7% |
| Simplified minus allele balance | 80.9% | 79.2% | 879 | 847 | 433 | 97.6% | 42.2% |
| Simplified minus  Alt allele depth | 82.3% | 92.6% | 886 | 896 | 377 | 96.3% | 64.4% |
| Simplified relaxed | 80.9% | 68.4% | 903 | 750 | 506 | 97.6% | 37.3% |
| Normalized allele dosage test > 4 | 82.3% | 93.1% | 919 | 884 | 356 | 96.3% | 66.1% |

Surprisingly, the simplified filter had about 5% higher specificity than the default tarSVM filter. Not using ExAC decreases the number of positive training examples by 12.4% as compared to the simplified filter, and increased the number of “unknown” examples by 76.3%. This resulted in an 8.5% reduction in the specificity of the SVM filter. Excluding the allele balance reduces the specificity by 29% as compared to the simplified filter. Removing the alt. allele depth reduces the specificity of the SVM by 6.8%. The simplified relaxed filter had nearly a 34% reduction in specificity. Finally, the normalized allele dosage test had the same performance as the standard tarSVM filter in terms of sensitivity and specificity of the SVM. These results lead us to conclude, not surprisingly, it is important to have a hard filter that is sensitive and specific, but it is also equally important to try to reduce the number of variants in the unknown category.

We then performed the same analysis using the CAKUT Cohort, and the results are in Table S2.

**Table S2: Influence of hard filtration on tarSVM for CAKUT** **Cohort**

| Filter | Hard filter  sensitivity | Hard filter  specificity | Number of positive examples | Number of negative  examples | Number of unknown examples | SVM  sensitivity | SVM  specificity |
| --- | --- | --- | --- | --- | --- | --- | --- |
| tarSVM standard | 59% | 87.3% | 1422 | 5772 | 1274 | 92.4% | 58.4% |
| Simplified | 51.1% | 87.8% | 1347 | 5929 | 1192 | 88.1% | 62.3% |
| Simplified minus ExAC | 72.7% | 96% | 946 | 5665 | 1857 | 93.1% | 47.6% |
| Simplified minus allele balance | 51.7% | 84.4% | 1686 | 2726 | 4056 | 91.2% | 55.1% |
| Simplified minus  Alt allele depth | 51.7% | 82.2% | 1411 | 5407 | 1650 | 91.2% | 44.8% |
| Simplified relaxed | 51.7% | 71.4% | 1629 | 3405 | 3434 | 95.6% | 30.2% |
| Normalized allele dosage test > 4 | 50.1% | 86.2% | 1396 | 5534 | 1538 | 91.2% | 57.5% |

Overall the standard tarSVM filter and the simplified filter perform similarly on this dataset. The standard tarSVM filter has 4.3% higher sensitivity, which is why we chose it as the default. We prefer an increased chance to find a variant and Sanger sequence a few extra false positive variants, rather than missing the true variant. Using ExAC improves the specificity by 15.8% but reduces the sensitivity by 5%. When looking at the variants reclassified by ExAC (Simplified filter versus Simplified minus ExAC), the ExAC database’s specificity is 70.1% and its sensitivity is 43.9%. Also, using ExAC decreases the proportion of variants classified as unknown by 35.8%. Overall this seems to assist the SVM with learning a decision boundary that increases specificity.

In conclusion, the standard tarSVM hard filter seems to perform more consistently across datasets than other filters. However, other filters that classify similar numbers of variants with similar specificity and sensitivity yield similar results as the standard tarSVM filter. A filter that does not assign enough negative training examples accurately drastically reduces the specificity of the SVM. The SVM is able to learn a more generalizable and accurate decision boundary as compared to the hard filters as evidenced by the large increases in sensitivity as compared to the hard filters in the CAKUT dataset.
