# Supplementary material for: tarSVM: Improving the accuracy of variant calls derived from microfluidic PCR-based targeted next generation sequencing using a support vector machine
Source: BMC Bioinformatics. 2016 Jun 10;17:233. doi: 10.1186/s12859-016-1108-4 (PMC4902911; doi:10.1186/s12859-016-1108-4)
Supplement: Additional file 4: — Supplemental Notes #2. (DOCX 50 kb) [file 12859_2016_1108_MOESM4_ESM.docx]

**Supplementary Note #2**: Influence of post-SVM genotype hard filter on overall variant classification in NS Cohort:

**Table S3**: Initial hard filter assignment from Step (A).

**Table S4**: SVM classification results from Step (C).

**Table S5**: Likely true variants from Step (A) in terms of SVM classification from Step (C)

**Table S6**: Likely false variants from Step (A) in terms of SVM classification from Step (C)

**Table S7**: Likely unknown variants from Step (A) in terms of SVM classification from Step (C)

**Table S8**: Results of Step (D) (post-SVM filtration)

**Supplementary Note #2: Influence of post-SVM genotype hard filter on overall variant classification in NS Cohort:**

tarSVM methodology has four steps: (A) Nine hard filters to classify variants into three groups (likely false, likely true, and unknown); (B) Training SVM using likely false and likely true variants; (C) Use trained SVM to make a prediction for all variants as “PASS” or “FILTERED”; and (D) Post-SVM filtration-apply a modest genotype level hard filter to remove likely false calls (see paper for specifics).

After step (A) for the NS Cohort, the variants are classified as shown in Table S3.

**Table S3: Initial hard filter assignment from Step (A).**

|  | Sanger Validated | Sanger  Not Validated | **Total** |
| --- | --- | --- | --- |
| Likely true | 58 | 2 | 60 |
| Likely false | 7 | 28 | 35 |
| Unknown | 18 | 29 | 47 |
| **Total** | 83 | 59 | 142 |

The sensitivity of variants with a prediction from the hard filtration is 58 / (58 + 7) = 89%. The specificity of variants with a prediction by the hard filtration is 28 / (2 + 28) = 93%. When we include unknown calls, the sensitivity is 58/83 = 70% and the specificity is 28 / 59 = 47%.

A SVM is trained using the variants classified the likely true and likely false variants from above (Step B), and we use the SVM to make a prediction for all variants (Step C). Table S4 has the results from this step (C).

**Table S4: SVM classification results from Step (C).**

|  | Sanger Validated | Sanger  Not Validated | **Total** |
| --- | --- | --- | --- |
| PASS | 80 | 20 | 100 |
| FILTERED | 3 | 39 | 42 |
| **Total** | 83 | 59 | 142 |

After applying the SVM, the sensitivity is 80 / 83 = 96%, and this specificity is 39 / (59) = 66%.

When comparing Step (A) to Step (C) for variants predicted as likely true by the hard filter (Table S5), we find that the SVM filter removed two variants that the hard filter predicted as true thus incurring a cost of two additional false negatives. For all other variants, both the SVM and hard filters agreed.

**Table S5: Likely true variants from Step (A) in terms of SVM classification from Step (C)**

|  | Sanger Validated | Sanger  Not Validated | **Total** |
| --- | --- | --- | --- |
| SVM PASS | 56 | 2 | 58 |
| SVM FILTERED | 2 | 0 | 2 |
| **Total** | 58 | 2 | 60 |

When comparing Step (A) to Step (C) for variants predicted to be likely false by the hard filters (Table S6), the SVM recovered six of seven variants that were Sanger validated but predicted as likely false by the hard filters. Interestingly, the sensitivity of the SVM using only variants that had a likely true or likely false classification is (56 + 6) / (58 + 7) = 95%, and the specificity is 27 / (2 + 28) = 90%. Thus the SVM had an increase in sensitivity, but a slight decrease in specificity as compared to the hard filters from Step (A).

**Table S6: Likely false variants from Step (A) in terms of SVM classification from Step (C)**

|  | Sanger Validated | Sanger  Not Validated | **Total** |
| --- | --- | --- | --- |
| SVM PASS | 6 | 1 | 7 |
| SVM FILTERED | 1 | 27 | 28 |
| **Total** | 7 | 28 | 35 |

When investigating variants that were predicted as unknown from Step (A), we find that 17 of 20 false positives (predicted by the SVM) are due to the “unknown” class of variants (Table S7), but no false negatives are coming from this group. The overall accuracy of variants from this is 30 / 47 or 64%. One may be tempted to conclude that the SVM has been overfit to the data, but the class of unknown variants are much harder to classify because they have many of the same characteristics of known variants found in ExAC or the 1000 Genomes, but many of them are in fact not true variants.

**Table S7: Likely unknown variants from Step (A) in terms of SVM classification from Step (C)**

|  | Sanger Validated | Sanger  Not Validated | **Total** |
| --- | --- | --- | --- |
| SVM PASS | 18 | 17 | 35 |
| SVM FILTERED | 0 | 12 | 12 |
| **Total** | 18 | 29 | 47 |

Finally in Step (D), the final genotype hard filter only reclassifies a single false positive variant from Step (C) as a true negative (Table S8). Thus, this step has a very marginal effect on the overall performance of tarSVM.

**Table S8: Results of Step (D) (post-SVM filtration)**

|  | Sanger Validated | Sanger  Not Validated | **Total** |
| --- | --- | --- | --- |
| PASS | 80 | 19 | 99 |
| FILTERED | 3 | 40 | 43 |
| **Total** | 83 | 59 | 142 |

In conclusion, the post-filtration step does not seem to have a major impact in either the NS cohort (showed above) or the CAKUT cohort (data not shown). However, the marginal improvement seen increases the specificity of the filter and this is why we chose to keep it. In addition, in principle, this step seems to be a reasonable filter to apply to improve our variant calling.
